# The effect of age on neural processing of pleasant soft touch stimuli

**DOI:** 10.3389/fnbeh.2014.00052

**Published:** 2014-02-21

**Authors:** April C. May, Jennifer L. Stewart, Susan F. Tapert, Martin P. Paulus

**Affiliations:** ^1^Department of Psychiatry, University of CaliforniaSan Diego, La Jolla, CA, USA; ^2^Psychiatry Service, Veterans Affairs San Diego Healthcare SystemSan Diego, CA, USA

**Keywords:** interoception, fMRI, development, CT afferents, reward, touch, adolescence, insular cortex

## Abstract

Tactile interactions with our environment stimulate afferent fibers within the skin, which deliver information about sensations of pain, texture, itch and other feelings to the brain as a comprehensive sense of self. These tactile interactions can stimulate brain regions involved in interoception and reward processing. This study examined subjective, behavioral, and neural processing as a function of age during stimulation of A-beta (Aβ) and C tactile (CT) afferents using a soft brush stroke task. 16 adolescents (ages 15–17), 22 young adults (ages 20–28), and 20 mature adults (ages 29–55) underwent a simple continuous performance task while periodically anticipating and experiencing a soft touch to the palm or forearm, during functional magnetic resonance imaging (fMRI). fMRI results showed that adolescents displayed greater bilateral posterior insula activation than young and mature adults across all conditions and stimulus types. Adolescents also demonstrated greater bilateral posterior insula activation than young and mature adults specifically in response to the soft touch condition. Adolescents also exhibited greater activation than mature adults in bilateral inferior frontal gyrus and striatum during the soft touch condition. However, mature adults showed greater striatum activation than adolescents and young adults during anticipation. In the left anterior cingulate cortex, mature adults exhibited greater activation than adolescents and young adults when anticipating the upcoming touch. These results support the hypothesis that adolescents show an exaggerated neural response to pleasant stimulation of afferents, which may have profound effects on how they approach or avoid social and risky situations. In particular, heightened interoceptive reactivity to pleasant stimuli might cause adolescents to seek experiences that are associated with pleasant stimulation.

## Introduction

Our sense of touch is important for how we interpret the world around us. Tactile interactions with our environment stimulate afferent fibers within the skin, which deliver information about sensations of pain, texture, itch, and other feelings from the body to the brain as a comprehensive sense of self (Craig, 2002; Olausson et al., 2002, 2010; Ackerley et al., 2012). Within human skin there are two types of mechanoreceptors known to respond to tactile stimulation: myelinated A-beta (Aβ) fibers and unmyelinated C tactile (CT) fibers. These two types of fibers are located in various regions throughout the body and respond to specific stimulation. Aβ fibers provide discriminate information about touch while CT afferents signal affective aspects of touch (Olausson et al., 2002; Bjornsdotter et al., 2010; Morrison et al., 2011b). Together, these afferents provide a comprehensive picture of tactile stimulation to regions of the brain. While previous research has investigated the neural response to tactile stimulation in adults (Olausson et al., 2002; Bjornsdotter et al., 2009) and age related differences in tactile stimulation between young and elderly individuals (Brodoehl et al., 2013), little is known about the specific developmental neural differences of these Aβ and CT afferent systems. The present study aims to investigate the functional development of brain regions involved in processing tactile stimulation via soft touch.

Within human skin there is a complex network of various types of afferent fibers, for review see (Abraira and Ginty, 2013), that each respond to specific sensations and together provide an overall representation of the physiological condition of the body (Craig, 2002; Olausson et al., 2002; Bjornsdotter et al., 2010). Aβ afferents are found in both the hairy skin of humans as well as glaborous skin, like that found on the palm. These Aβ afferents respond to most kinds of mechanical stimulation and provide discriminative information about the touch sensation (Bjornsdotter et al., 2010). CT afferents, on the other hand, are found strictly in hairy human skin present in the forearm and preferentially respond to types of pleasant touch often experienced during social interactions, specifically a gentle stroking at a velocity of 1–10 cm/s (Loken et al., 2009; Morrison et al., 2011a). As such, CT afferents are hypothesized to encode the rewarding and emotional properties of touch (Olausson et al., 2002; Bjornsdotter et al., 2009, 2010; Morrison et al., 2011b; McGlone et al., 2012). CT afferents carry this information about the physiological condition of the body from the periphery to the thalamus, delivering sensory information on to the posterior insula and then to the left anterior insula for integration (Olausson et al., 2002; Craig, 2003; Bjornsdotter et al., 2009; Loken et al., 2009; Paulus and Stein, 2010; McGlone et al., 2012) contributing to an overall awareness of the body's condition by providing signals of the experienced sensation (Craig, 2002; Naqvi and Bechara, 2009; Paulus et al., 2009; Bjornsdotter et al., 2010). Although CT afferents mainly provide hedonic information, Aβ afferents can also produce a pleasant sensation as demonstrated by a soft brush stroke on the palm, in an area where CT afferents are lacking (Kramer et al., 2007). In addition, Aβ afferents have been shown to activate insular connections to regions of orbitofrontal cortex involved in emotional evaluation (Olausson et al., 2010). Investigating the neural response to soft touch in adolescents could provide a better understanding of how adolescents perceive and interpret interoceptive and rewarding aspects of social pleasant touch.

Many psychiatric disorders emerge clinically during adolescence, a time marked by significant physical and behavioral changes (Kessler et al., 2007; Paus et al., 2008). Among these are psychiatric conditions associated with increased risk taking behavior, e.g., experimentation with drugs and alcohol (Bjork et al., 2004). One explanation for these marked differences could be that adolescents engage in these behaviors and seek to administer drugs or alcohol because they have an altered, undeveloped, or over-reactive interoceptive regulatory system. It has been proposed that the difference between the expected and observed body state provides a learning signal, known as a body prediction error, that motivates an individual to adjust their behavior in order to maintain homeostasis (Paulus et al., 2009). Few studies have examined developmental differences in brain regions involved in interoception. In adolescents, poor interoceptive awareness has been linked to the development of eating disorders (Keel et al., 1998), whereas heightened interoceptive awareness has been linked to panic disorder (Hoffman and Mattis, 2000; Paulus and Stein, 2006). Prior work has also shown that interoceptive awareness declines with age in a sample ranging from 22–63 years (Khalsa et al., 2009). However, interoceptive development has not yet been investigated within the specific context of probing responses to pleasant and rewarding touch in healthy individuals.

Pleasant touch has also been shown to activate regions of the prefrontal cortex, such as the orbitofrontal cortex, because of its role in processing reward and assessing hedonic valence (Craig, 2002; McCabe et al., 2008; Gordon et al., 2013; Voos et al., 2013). In adolescents, the prefrontal cortex is known to be underdeveloped, leading to immature cognitive control abilities and contributing to increased risk-taking (Van Leijenhorst et al., 2010). Previous research found the orbitofrontal cortex to activate more in response to soft touch on the forearm, as opposed to soft touch on the palm, because the CT afferents found in the forearm are thought to underlie emotional responses to light soft touch. This research also found activation in prefrontal regions to correlate with subjective ratings of pleasantness (McCabe et al., 2008). When experiencing soft touch, the foundation for our subjective interpretation of our bodily state is developed in the insula and relayed to the orbitofrontal cortex (Craig, 2002). Understanding the response of the insular cortex and prefrontal regions in adolescents when exposed to pleasant touch could expand our understanding of how adolescents interpret and respond to pleasant stimulation.

Adolescents perceive less threat from potential risks, and as such, they believe risky outcomes to be more in their control than do adults (Benthin et al., 1993). Previous studies have shown the dorsal anterior cingulate cortex (ACC) to be active when anticipating events and encoding rewarding values, specifically during reward-based decision making (Bush et al., 2002). Adults were shown to have overall greater bilateral dorsal ACC activation than adolescents when making risky choices, suggesting the ACC plays a greater role in behavioral control for adults than adolescents (Eshel et al., 2007). Another explanation for the prevalence of risk taking during adolescence may be an overactive ventral striatal response to rewarding stimuli (Bjork et al., 2004), which could contribute to reward seeking and experimentation with drugs and alcohol (Cohen et al., 2010). Therefore, through tactile stimulation, the present investigation seeks to provide novel information on the development of adolescent brain regions implicated in interoceptive and reward processing to gain understanding of typical development, as well as atypical development, which could signal future onset of behavioral and emotional problems.

The goal of this study was to determine whether there are developmental differences in the processing of pleasant soft touch in the insular cortex, ventral striatum, dorsal ACC, and regions of the prefrontal cortex. Based on the increased susceptibility to psychiatric conditions that affect reward-related processing in adolescence, we predicted that adolescents exhibit an exaggerated response to the processing of pleasant stimuli. We investigated this issue by examining the relationship between age and neural activation during a basic attention task coupled with the administration of a soft touch to the palm and forearm, thereby stimulating both Aβ and CT afferents. Because both Aβ and CT afferents have been shown to activate regions of the insular cortex (Craig, 2003; Olausson et al., 2010) the present study aims to investigate changes in insular activation as a function of age. Four primary hypothesis were tested. First, consistent with evidence demonstrating that interoceptive awareness decreases with age (Khalsa et al., 2009), we hypothesized that adolescents (AD) will exhibit greater activation in the posterior insula than young adults (YA) and mature adults (MA), and therefore, age will negatively correlate with posterior insular activation. Second, because we expect to find a negative correlation between age and posterior insula activation, we hypothesized that AD, relative to MA, will also display greater activation in prefrontal regions, as these regions are responding to the evaluation provided by the insula. Third, in line with previous research, we hypothesized that MA would exhibit greater dorsal ACC activation during the anticipation of upcoming rewards than AD and YA. Lastly, consistent with evidence demonstrating increased ventral striatum response in AD, highlighting their heightened sensitivity to the rewarding properties of stimuli (Bjork et al., 2004) we predicted that AD would display heightened striatal activation in response to the rewarding soft touch.

## Methods

### Subjects

A total of 58 healthy subjects ranging from ages 15–55 completed clinical assessments in addition to functional magnetic resonance imaging (fMRI) recording while engaged in a continuous performance task (CPT). Participants were categorized into one of three age groups: 16 AD ages 15–17, 22 YA ages 20–28 and 20 MA ages 29–55. These age ranges were defined according to Bjork et al. (2004) who had investigated reward-related brain processing differences across adolescents and adults. Moreover, in order to determine that our group definition, although based on prior work, was not inducing an arbitrary finding, we also conducted a dimensional analysis with age as our independent variable and activation during soft touch as our dependent measure. The University of California, San Diego Human Research Protections Program and the Veterans Affairs San Diego Healthcare System Research and Development Office approved the study protocol. Written informed consent was obtained from all participants prior to enrollment in the study. Participants were recruited through local high schools, universities and the general public (e.g., flyers, Craigslist).

All participants underwent a detailed clinical interview using the Semi-Structured Assessment for Drug Dependence and Alcoholism (SSADDA) (Koob and Le Moal, 2001) to confirm the absence of clinical diagnoses. To meet inclusion criteria, all participants endorsed no current or lifetime history of DSM-V (American Psychiatric Association, 2013) substance use disorder (except nicotine), psychotic disorder, or antisocial personality disorder, and no current mood, anxiety or attention disorders according to structured interview. In addition, participants could not have any of the following: (1) a current severe medical disorder requiring inpatient treatment; (2) pregnancy; (3) left handedness; and (4) head injuries or loss of consciousness >5 min. Absence of DSM-V diagnoses were presented at a consensus meeting and confirmed by a psychiatrist (MPP).

At the time of the clinical interview, participants completed personality measures including the Barratt Impulsiveness Scale (BIS) (Patton et al., 1995) and the Sensation seeking Scale form V (SSS-V) (Zuckerman, 1996) as measures of impulsivity and sensation seeking, respectively.

### Soft touch task

The soft touch stimulus consisted of a light brush stroke administered by trained research assistants using a hand held soft boar bristle brush (OXO International Ltd., NY). The soft touch was administered at a velocity of 2 cm/s (Loken et al., 2009) in a proximal to distal direction with a force equal to the weight of the brush on pre-measured and marked 4 cm long regions of skin on the ventral surface of the left forearm, thought to be dense in Aβ and CT afferents, and on the palm, which only contains Aβ afferents (Vallbo et al., 1993; Olausson et al., 2000; Loken et al., 2009).

**Figure 1:**
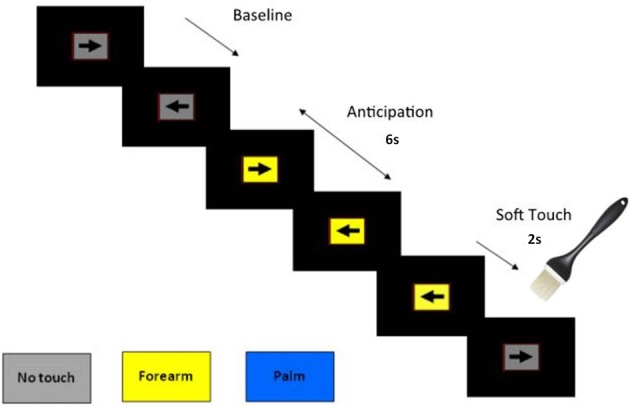
**Illustration of Soft Touch continuous performance task**.

During the fMRI session, participants performed a CPT with cued stimulus presentation, which was designed to focus their attention on the visual stimuli while maintaining a stable cognitive load. In addition, the CPT was used to determine if the soft touch stimulus distracted the participants from the task at hand. For the CPT, a screen presented a left or right pointing black arrow surrounded by a colored rectangle in successive 3 s intervals (see Figure 1). Subjects were instructed to respond to the orientation of the arrow by pressing a left or right button on a button box. The arrow remained on the screen for the entire 3 s during which the subject could press a button at any time. The colored rectangle background was used to signify one of three conditions: (1) a *baseline condition* (gray background) during which no tactile stimulus was expected or administered, with variable duration averaging 8 s; (2) an *anticipation condition* lasting 6 s during which the background color of the presentation indicated an impending soft touch on the left palm (blue background) or left forearm (yellow background); (3) a *Soft Touch condition* lasting 2 s during which a soft touch to the skin was administered to either the palm or the forearm for a total of 20 repetitions each. Total task duration was 840 s split between two runs of 420 s each. The task was administered across two runs in order to give participants a short break and prevent fatigue.

Response accuracy and reaction time (RT) were obtained during all conditions. Participants received instruction on task structure and background color meanings prior to fMRI acquisition and completed post-fMRI visual analog scale (VAS) questionnaires. VAS instructions indicated that participants should provide a rating of pleasantness from “not at all” to “extremely” about their experience of the Soft Touch task.

### fMRI data acquisition

The Soft Touch task was conducted during two fMRI runs sensitive to blood oxygenation level-dependent (BOLD) contrast using a Signa EXCITE (GE Healthcare, USA) 3.0 Tesla scanner (T2^*^-weighted echo planar imaging (EPI) scans, *TR* = 2000 ms, *TE* = 32 ms, *FOV* = 24 cm (squared), 64 × 64 matrix, thirty 2.6 mm axial slices with a 1.4 mm gap, flip angle = 90°, 210 whole-brain acquisitions). For anatomical reference, a high-resolution T1-weighted image [spoiled gradient recalled (SPGR), *TR* = 8 ms, *TE* = 3 ms, slices = 172, *FOV* = 25 cm approximately 1 mm (cubed) voxels] was obtained. The CPT was presented in an event related design.

### fMRI data analysis

#### Single subject analysis

fMRI data were preprocessed with the Analysis of Functional NeuroImages (AFNI) software package (Cox, 1996). GE slices were first reconstructed into AFNI BRIK format. The largest temporal region containing the fewest voxel-wise outliers was used as a base for 3d registration. All other time points in dx, dy, dz, and roll, pitch, yaw directions were adjusted to align data to the base image. The functional echoplanar image underwent automatic coregistration to the high-resolution anatomical image and each dataset was inspected to confirm successful alignment. New outliers were generated for the volume-registered dataset based on whether a given time point greatly exceeded the mean number of voxel outliers for the time series. Deconvolution was performed to determine Soft Touch decision phase activations. Three movement regressors (roll, pitch, yaw), a baseline and linear drift regressor, and four decision-making regressors (trials for anticipation palm, anticipation forearm, soft touch palm, soft touch forearm), were convolved with a modified hemodynamic response function. The baseline condition, wherein participants were neither anticipating nor receiving the soft touch stimulus, served as the baseline for this analysis. A Gaussian Spatial Filter (4 mm full width-half maximum) was used to spatially blur data to account for anatomical differences. Automated Talairach transformations were applied to anatomical images and echoplanar images were subsequently transformed into Talairach space. Percent signal change was determined by dividing the signal for each regressor of interest by the baseline regressor.

#### Group analysis

A linear mixed effects (LME) analysis (r-project.org) was performed to examine group differences in brain activation. Subjects were treated as random effects while group (AD, YA, MA), condition (anticipation, soft touch), and stimulus type (palm, forearm) were treated as fixed effects. Percent signal change from baseline was the dependent variable. The main effect of group was examined to determine whether age groups differed across conditions and stimulus types. The group by condition interaction was the primary effect of interest in order to test hypotheses involving anticipation and receipt of pleasant touch in AD, YA, and MA. To guard against identifying false positive areas of activation, a threshold adjustment method based on Monte-Carlo simulations (via AFNI AlphaSim program) was applied. For the whole brain analysis, AlphaSim identified a minimum cluster volume of 512 μL (8 contiguous voxels) corresponding to a cluster significance of *p* < 0.01 (one-sided) to result in a voxel-wise probability of *p* < 0.01 (one-sided) corrected for multiple comparisons. In addition, to further examine the role of brain regions involved in *a priori* hypotheses, restricted regions of interest masks for the insula, ACC and striatum were applied to LME results. These masks were defined by the Talairach atlas (Lancaster et al., 2000) and AlphaSim identified a minimum cluster volume of 256 μL (4 contiguous voxels) for bilateral insula and ACC and a minimum cluster of 192 μL (3 contiguous voxels) for bilateral striatum (all *p* < 0.01 corrected). In addition, *post-hoc t*-tests were computed for results from each region of interest (ROI) to determine which groups significantly differed from one another.

#### Dimensional analysis

Activation found in apriori regions of interest from the group by condition interaction were also correlated dimensionally with age across all participants to examine whether interoceptive awareness changes as a function of development. Both Pearson and Spearman correlation analyses were performed.

### Self-report/behavioral data analysis

A univariate analysis of variance (ANOVA) analysis was performed to investigate group differences in VAS ratings of soft touch, with group (AD, YA, MA) as the between subject variable and VAS ratings of pleasantness as the dependent measure. Correlations were also computed to investigate the relationship between age and pleasantness. A repeated measures ANOVA was also performed to investigate RT differences with the baseline condition subtracted, wherein soft touch stimulus type (forearm, palm) was the within-subjects variables and group (AD, YA, MA) was the between subjects variable.

## Results

### Subject characteristics

By definition, AD, YA, and MA significantly differed in age as well as education, but groups did not differ in verbal IQ, ethnicity, gender or BIS ratings (see Table 1). YA reported significantly higher SSS ratings than AD but neither group differed from MA.

**Table 1:** **Subject characteristics by group**.

	**Adolescents (AD)**		**Young adults (YA)**		**Mature adults (MA)**		***df***	**F/X^2^**	***P***
	***N*** = **16**		***N*** = **22**		***N*** = **20**				
**CHARACTERISTICS**	**Mean**	***SD***	**Mean**	***SD***	**Mean**	***SD***			
Age	16.63	0.50	24.10	1.9	37.75	8.25	2, 55	84.42	<0.001
Education	10.69	0.48	15.36	1.53	15.30	1.63	2, 55	66.96	<0.001
Verbal IQ	112.31	14.43	115.74^†^	9.72	109.05^†^	11.19	2, 51	1.53	0.23
**DEMOGRAPHICS**	***N***	**%**	***N***	**%**	***N***	**%**			
Female	7	43.8	14	63.6	6	30	2	4.83	0.09
Caucasian	15	93.8	16	72.7	15	75	4	8.74	0.07
Hispanic	2	12.5	6	27.3	5	25	2	1.28	0.53
**QUESTIONNAIRES**	**Mean**	***SD***	**Mean**	***SD***	**Mean**	***SD***			
Barratt impulsivity scale	57.00	11.64	56.63^†^	11.58	52.85	7.59	2, 52	0.94	0.40
Sensation seeking scale	14.94	3.91	19.95^†^	4.24	17.63^†^	8.04	2, 51	3.24	0.05
**VAS RATINGS**									
Pleasant	4.80	2.11	5.54	2.01	5.33	2.33	2, 55	0.57	0.57
Intensity	1.16	1.14	0.88	0.94	1.45	1.63	2, 55	1.03	0.36

† n = 19.

### Behavioral data

#### VAS ratings

AD, YA, and MA did not differ in their subjective ratings of the pleasantness of the soft touch (See Table 1).

#### RT and accuracy

When accounting for baseline, groups did not differ in RT. However, across participants, RT was significantly slower during the soft touch to the palm than the soft touch to the forearm. All groups performed similarly with no significant differences in accuracy across conditions and stimulus types (See Table 2).

**Table 2:** **Behavioral data by group**.

	**Adolescents (AD)**		**Young adults (YA)**		**Mature adults (MA)**	
	*****N*** = **16****		***N*** = 19^*^		***N*** = 19^*^	
**REACTION TIME**	***M***	***SD***	***M***	***SD***	***M***	***SD***
Baseline	613.99	110.77	714.76	152.37	855.09	270.88
Anticipation palm	627.15	95.98	676.26	136.12	812.40	241.33
Anticipation forearm	620.17	109.89	690.35	167.28	836.83	330.97
Soft touch palm	649.01	145.43	717.41	204.51	892.69	356.36
Soft touch forearm	633.62	152.92	680.78	179.50	837.59	330.92
**ANOVA FOR REACTION TIME**	***F*(*df*)**	***P***			***F*(*df*)**	***P***
Group	1.90 (2, 51)	0.159	Group by condition		0.397 (2, 51)	0.674
Condition	3.68 (1, 51)	0.061	Group by stimtype		0.026 (2, 51)	0.974
Stimtype	2.15 (1, 51)	0.149	Condition by stimtype		4.53 (1, 51)	0.038
			Group by condition by stimtype		0.872 (2, 51)	0.424
**ACCURACY**	**M**	***SD***	**M**	***SD***	**M**	***SD***
Baseline	0.993	0.006	0.950	0.214	0.980	0.061
Anticipation palm	0.991	0.019	0.999	0.006	0.966	0.127
Anticipation forearm	0.996	0.008	0.998	0.008	1.00	0.000
Soft touch palm	0.996	0.015	1.00	0.000	0.971	0.090
Soft touch forearm	0.999	0.013	1.00	0.000	0.976	0.079
**ANOVA FOR ACCURACY**	***F*(*df*)**	***P***			***F*(*df*)**	***P***
Group	1.23 (2, 51)	0.300	Group by condition		2.44 (2, 51)	0.097
Condition	0.239 (1, 51)	0.627	Group by stimtype		1.26 (2, 51)	0.292
Stimtype	1.57 (1, 51)	0.216	Condition by stimtype		0.852 (1, 51)	0.360
			Group by condition by stimtype		0.708 (2, 51)	0.497

* Reaction time and accuracy data are missing for 3 YA and 1 MA due to recording error.

### fMRI data

#### Group main effect

**Figure 2:**
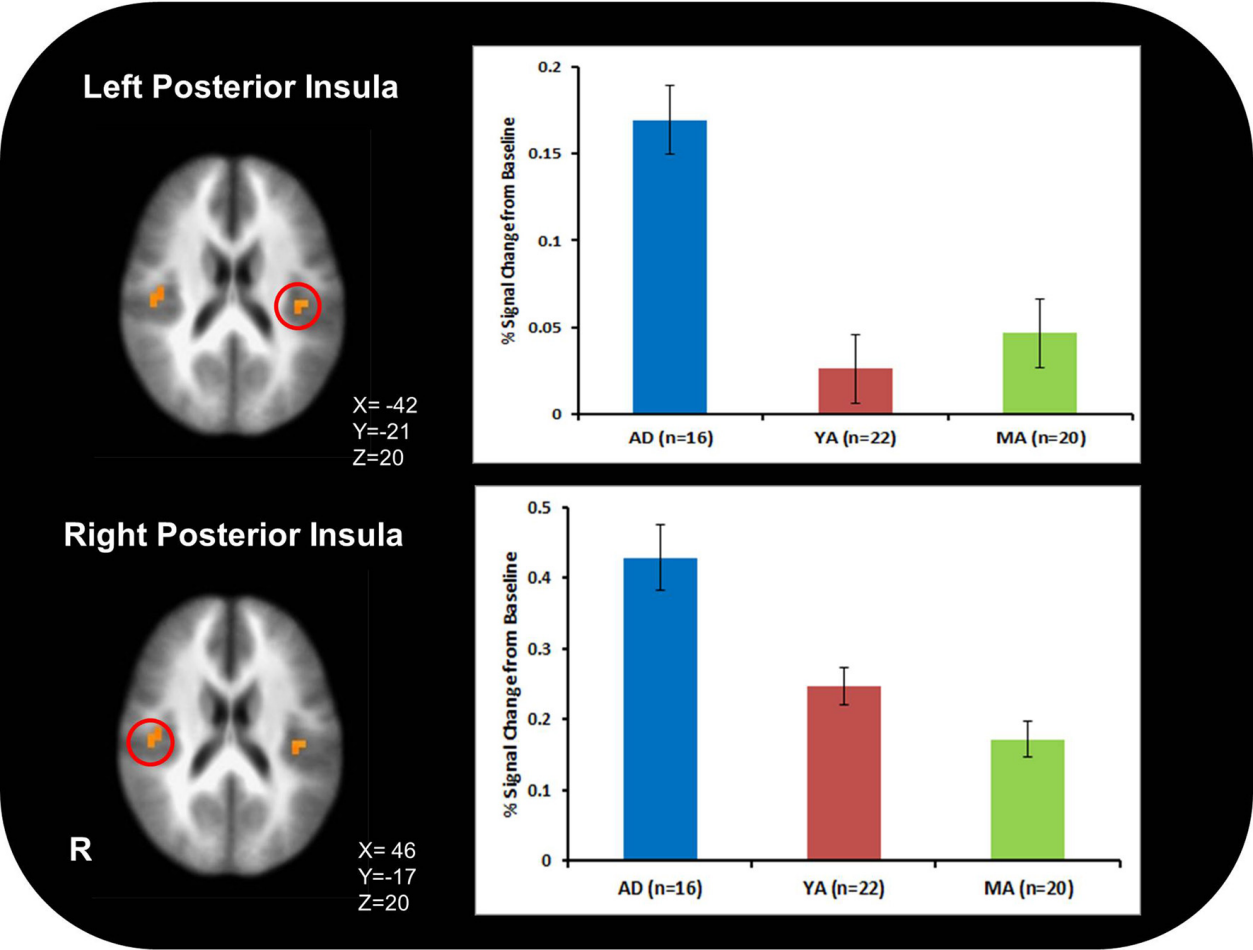
**Adolescents (AD) exhibited greater bilateral posterior insula activation than young adults (YA) and mature adults (MA) across all conditions and stimulus types**.

AD exhibited greater activation than YA and MA in bilateral posterior insula across all conditions and stimulus types (Figure 2; Table 3).

**Table 3:** **fMRI results for main effect of group**.

**Mask**	**No. of voxels in cluster**	**Volume (μL)**	***x***	***y***	***z***	**L/R**	**Center of mass**	**Relationship**
Whole brain	113	7232	−2	−53	26	L	Cingulate gyrus	AD>YA=MA
Whole brain	34	2176	−27	−50	61	L	Superior parietal lobule	AD>YA=MA
Whole brain	31	1984	−32	−52	−16	L	Culmen	AD=MA>YA
Whole brain	31	1984	57	−10	−8	R	Middle temporal gyrus	AD>MA>YA
Whole brain	29	1856	−50	−35	46	L	Inferior parietal lobule	AD>YA=MA
Whole brain	28	1792	26	−22	66	R	Precentral gyrus	AD>YA=MA
Whole brain	22	1408	−42	−68	5	L	Middle occipital gyrus	AD>YA=MA
Whole brain	22	1408	−46	−23	24	L	Inferior parietal lobule	AD>YA=MA
Whole brain	17	1088	22	−55	61	R	Superior parietal lobule	AD>YA=MA
Whole brain	15	960	−22	−9	2	L	Lentiform nucleus	AD=YA>MA
Whole brain	13	832	−12	−34	−2	L	Parahippocampal gyrus	AD=MA>YA
Whole brain	13	832	44	−71	2	R	Inferior temporal gyrus	AD>YA=MA
Whole brain	12	768	18	1	−17	R	Parahippocampal gyrus	AD>YA=MA
Whole brain	12	768	21	−64	−7	R	Fusiform gyrus	AD>YA=MA
Whole brain	12	768	50	−15	7	R	Superior temporal gyrus	AD=MA>YA
Whole brain	12	768	16	−22	12	R	Thalamus	AD=MA>YA
Whole brain	12	768	−23	−30	55	L	Postcentral gyrus	MA>AD=YA
Whole brain	11	704	−10	−49	−15	L	Culmen	MA>AD=YA
Whole brain	10	640	58	−44	25	R	Inferior parietal lobule	AD=MA>YA
Whole brain	9	576	57	−19	−16	R	Inferior temporal gyrus	AD>YA=MA
Whole brain	9	576	20	−96	4	R	Cuneus	AD>YA=MA
Whole brain	8	512	−35	−77	18	L	Middle temporal gyrus	AD=MA>YA
Whole brain	8	512	53	−53	21	R	Superior temporal gyrus	AD=MA>YA
rROI	5	320	44	−15	7	R	Posterior insula	AD=MA>YA
rROI	4	256	−2	14	−8	L	Anterior cingulate	AD>YA=MA
rROI	4	256	−39	−22	19	L	Posterior insula^2^	AD>YA=MA
rROI	4	256	44	−17	20	R	Posterior insula^2^	AD>YA=MA

AD, adolescents; YA, young adults; MA, mature adults; L, left hemisphere; R, hemisphere; rROI, Restricted ROI based on hypothesis. Talairach coordinates reflect center of mass.

2 Figure 2.

#### Group by condition interaction

**Figure 3:**
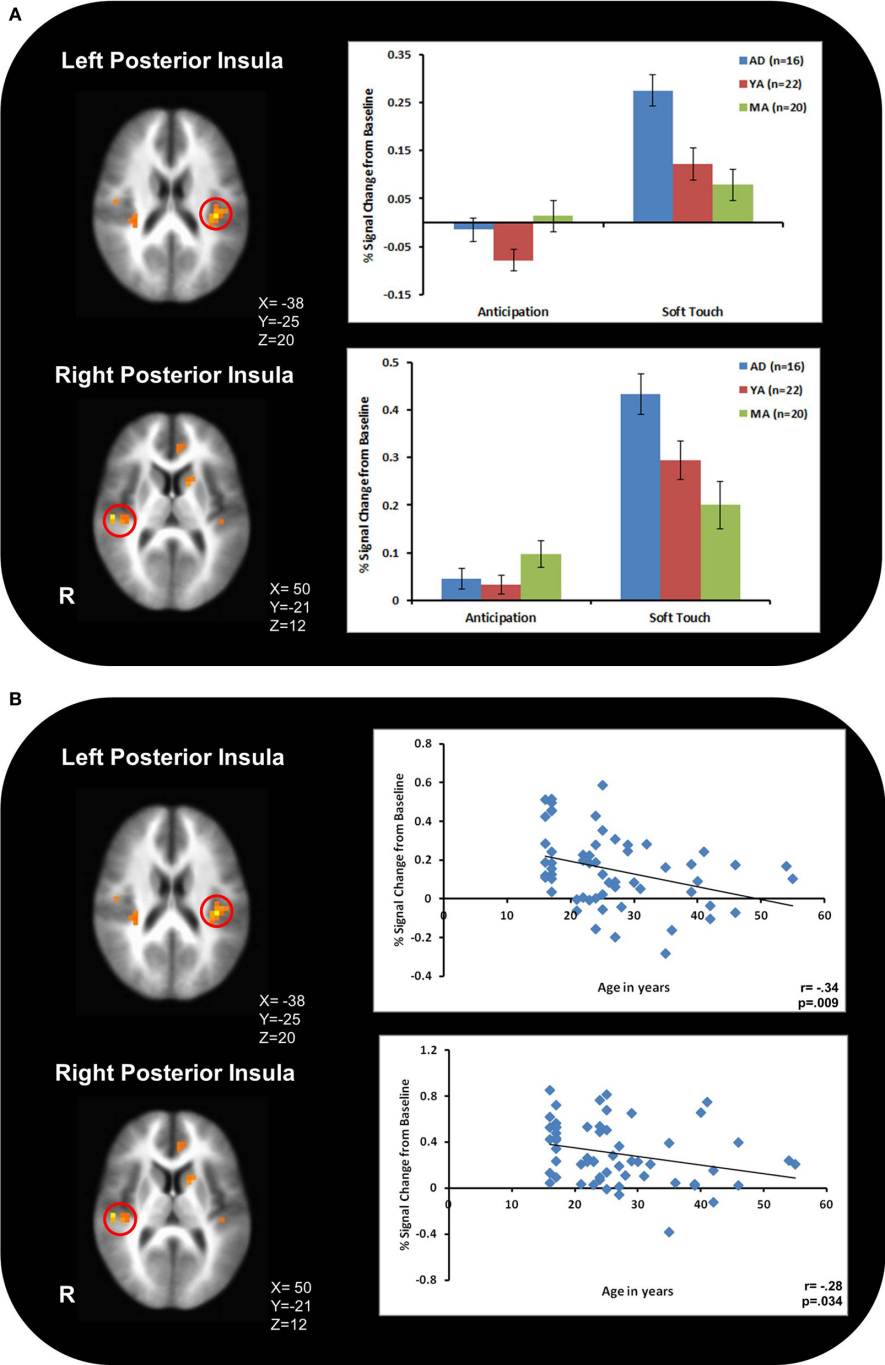
**(A)** During the soft touch condition, adolescents, (AD) displayed greater activation than young adults (YA) and mature adults (MA) in bilateral posterior insula. **(B)** The relationship between age and bilateral posterior insula activation showed a significant negative correlation.

**Figure 4:**
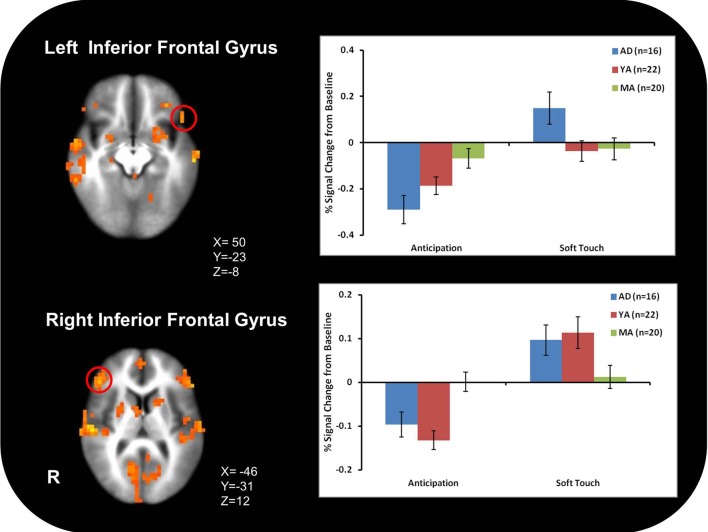
**Adolescents (AD) showed greater activation than mature adults (MA) in bilateral inferior frontal gyrus (IFG)**. However, young adults (YA) showed deactivation in left IFG and activation comparable to adolescents (AD) in right IFG.

**Figure 5:**
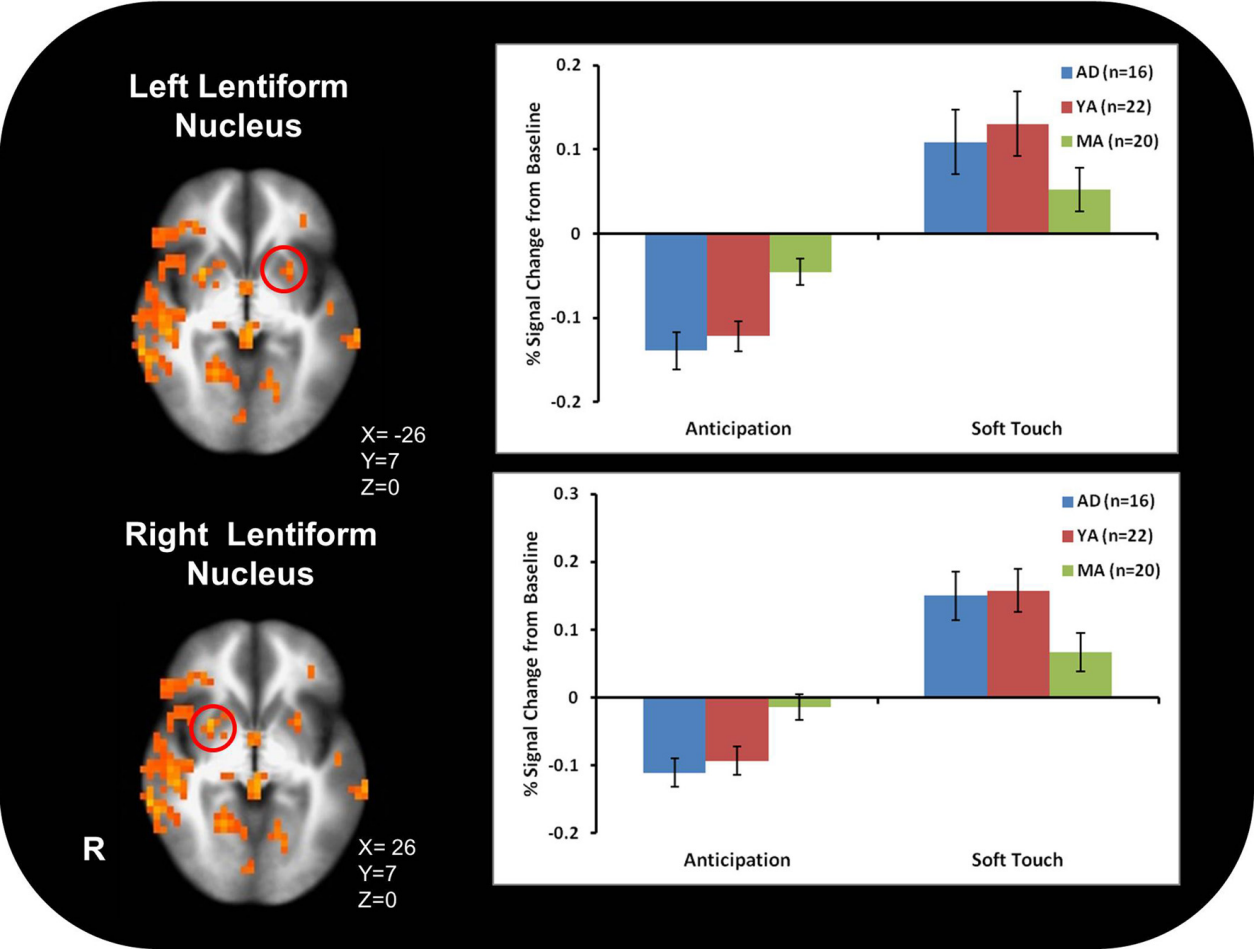
**In bilateral ventral striatum (Ientiform nucleus), mature adults (MA) showed greater activation than adolescents (AD) and young adults (YA) during anticipation**. During the Soft touch condition (AI) and YA showed greater activation than MA in the right ventral striatum.

**Figure 6:**
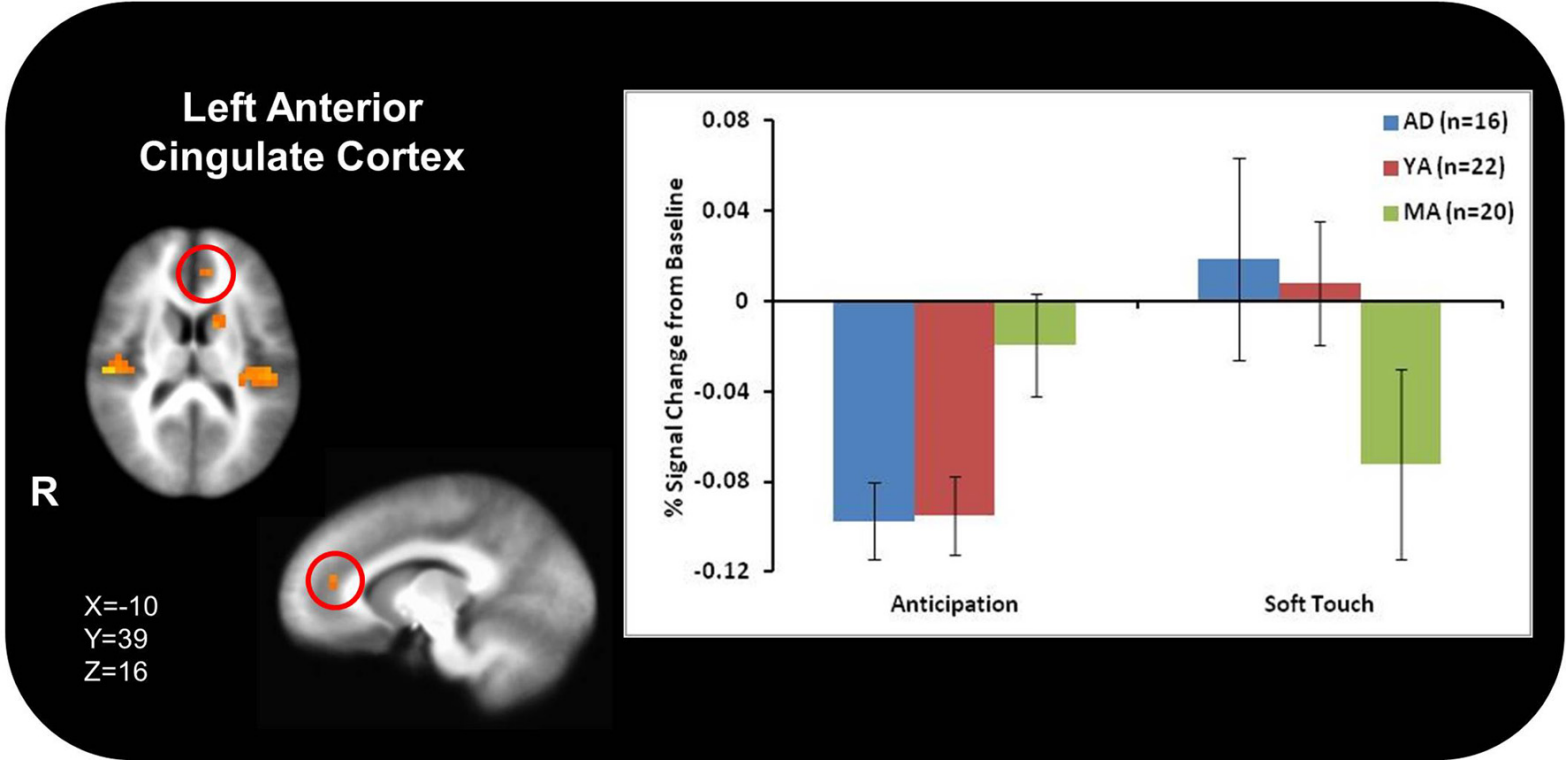
**In the left dorsal anterior cingulate (ACC), adolescents (AD) and young adults (YA) displayed comparable deactivation during anticipation, where as during the soft touch they displayed heightened activation**. In contrast, mature adults (MA) exhibited comparable attenuation during both anticipation and soft touch.

Figure 3A illustrates that AD displayed greater activation than YA and MA in bilateral posterior insula during soft touch but not anticipation. In addition, AD showed heightened activation in bilateral inferior frontal gyrus (IFG) greater than MA during the soft touch. YA however, showed deactivation in left IFG comparable to MA, yet activation in right IFG comparable to AD (Figure 4; Table 4). Although MA showed greater bilateral striatum (lentiform nucleus) activation than AD and YA during anticipation, AD and YA showed greater activation than MA during soft touch (Figure 5). Lastly, although groups did not differ in dorsal ACC activation during soft touch, MA exhibited greater activation than both AD and YA when anticipating the upcoming soft touch stimulus (Figure 6).

**Table 4:** **fMRI results for group by condition interaction**.

**Mask**	**No. of voxels in cluster**	**Volume (μL)**	***x***	***y***	***z***	**L/R**	**Center of mass**	**Anticipation**	**Soft touch**
Whole brain	783	50112	25	−28	41	R	Cingulate gyrus±	MA>AD=YA	AD>MA
Whole brain	326	20864	0	−67	16	L	Posterior cingulate	MA>YA	AD>MA
Whole brain	153	9792	41	31	11	R	Inferior frontal gyrus^4^	MA>AD=YA	AD=YA>MA
Whole brain	81	5184	−52	−22	10	L	Transverse temporal gyrus	MA>AD=YA	AD>MA
Whole brain	74	4736	−2	48	30	L	Superior frontal gyrus	MA>YA=AD	AD>MA
Whole brain	53	3392	22	32	44	R	Superior frontal gyrus	MA>AD=YA	AD>MA
Whole brain	47	3008	22	5	4	R	Lentiform nucleus^5^	MA>AD=YA	AD=YA=MA
Whole brain	44	2816	39	8	−29	R	Superior temporal gyrus	AD=YA=MA	AD=YA>MA
Whole brain	32	2048	42	12	32	R	Middle frontal gyrus	AD=MA>YA	AD=YA=MA
Whole brain	26	1664	−46	33	14	L	Inferior frontal gyrus	MA>AD=YA	AD>MA
rROI	26	1664	−40	−25	18	L	Posterior insula^3a^	AD=YA=MA	AD>YA=MA
Whole brain	25	1600	−25	5	−11	L	Subcallosal gyrus	MA>YA	AD>MA
Whole brain	25	1600	−5	13	37	L	Cingulate gyrus	MA>AD=YA	AD=YA=MA
Whole brain	23	1472	−33	21	42	L	Middle frontal gyrus	MA>YA	AD=YA=MA
Whole brain	22	1408	−20	8	8	L	Lentiform nucleus^5^	MA>AD=YA	AD=YA=MA
Whole brain	22	1408	14	−1	63	R	Superior frontal gyrus	MA>YA	YA>MA
Whole brain	21	1344	47	−63	27	R	Middle temporal gyrus	MA>YA	AD>MA
Whole brain	21	1344	−28	−5	55	L	Middle frontal gyrus	AD=YA=MA	MA=YA>AD
rROI	19	1216	45	−20	14	R	Posterior insula^3a^	AD=YA=MA	AD>MA
Whole brain	18	1152	1	−2	6	R	Thalamus	AD=YA=MA	AD=YA=MA
Whole brain	15	960	33	−57	56	R	Superior parietal lobule	AD=YA=MA	YA>MA
rROI	15	960	45	11	2	R	Anterior insula	MA>AD=YA	AD=YA=MA
Whole brain	13	832	−63	−14	−10	L	Middle temporal gyrus	MA>YA	AD>MA
Whole brain	13	832	−16	36	43	L	Superior frontal gyrus	MA>AD	AD=YA=MA
Whole brain	11	704	−47	15	−23	L	Superior temporal gyrus	MA>YA	AD>YA=MA
Whole brain	11	704	−9	−14	16	L	Thalamus	MA=AD>YA	AD=YA=MA
Whole brain	10	640	−12	−31	−16	L	Culmen	AD=YA=MA	MA>AD=YA
Whole brain	10	640	−34	37	−5	L	Middle frontal gyrus	MA>AD=YA	AD>MA
Whole brain	10	640	−48	−15	42	L	Precentral gyrus	MA>AD=YA	AD=YA=MA
Whole brain	9	576	20	−24	−4	R	Parahippocampal gyrus	AD=YA=MA	AD>YA=MA
Whole brain	8	512	−49	23	−6	L	Inferior frontal gyrus^4^	MA>AD	AD>YA=MA
rROI	7	448	43	−19	0	R	Posterior insula	AD=MA>YA	AD>YA=MA
rROI	5	320	−8	38	14	L	Anterior cingulate^6^	MA>AD=YA	AD=YA=MA
rROI	4	256	31	−29	20	R	Posterior insula	AD=YA=MA	AD>MA

AD, adolescents; YA, young adults; MA, mature adults; L, left hemisphere; R, right hemisphere; rROI, Restricted ROI based on hypothesis. Talairach coordinates reflect center of mass.

3a Figure 3;

4 Figure 4;

5 Figure 5;

6 Figure 6.

± Region also includes posterior insula, postcentral gyrus, precentral gyrus, superior temporal gyrus.

#### Dimensional analysis

Table 5 presents the findings for correlations between activation during the soft touch condition in a-priori regions of interest (posterior insula, IFG, lentiform nucleus, left dorsal ACC) and age. Age was also correlated with activation in left dorsal ACC during both the anticipation condition. Bilateral posterior insula showed a significant negative correlation with age. In addition, the right IFG and right lentiform nucleus showed a marginal negative correlation with age. Specifically, older relative to younger individuals showed attenuated brain activation during soft touch stimulation.

**Table 5:** **ROI activation from group by condition analyses correlated with age**.

	**Pearson (r)**	**Spearman (p)**
**SOFT TOUCH**		
Left posterior insula	−0.34^*^	−0.37^*^
Right posterior insula	−0.28^*^	−0.32^*^
Left inferior frontal gyrus	−0.19	−0.21
Right inferior frontal gyrus	−0.23^†^	−0.24^†^
Left lentiform nucleus	−0.17	−0.15
Right lentiform nucleus	−0.25^†^	−0.23^†^
Left anterior cingulate cortex	−0.33^*^	−0.18
**ANTICIPATION**		
Left anterior cingulate cortex	0.33^*^	0.20

* Significant at p < 0.05.

† Marginally significant at p < 0.10.

## Discussion

This study examined four hypothesis regarding developmental differences in neural processing of soft touch. Consistent with our first prediction, AD exhibited greater activation in the posterior insula, specifically in response to the experience of the soft touch. In line with our second hypothesis, AD showed greater activation than MA in bilateral inferior frontal gyrus. Third, in line with our hypothesis, MA showed greater activation than YA in the left dorsal ACC when anticipating upcoming rewards. Fourth, while we hypothesized that AD would exhibit greater striatal activation during the soft touch than YA and MA this was not the case. AD and YA showed comparable activation to one another greater than MA but this difference was only statistically significant in the right lentiform nucleus. Lastly, in order to examine whether the effect age was significant if considered as a continuous variable, we conducted correlational analyses which showed a negative correlation between age and posterior insula activation, suggesting that activation in posterior insula declines with age. Taken together, relative to MA, AD appear to have an exaggerated neural reactivity to pleasant stimuli in various regions implicated in interoception and reward processing.

These findings of developmental differences in insular response support the notion of an exaggerated interoceptive response in adolescents. Results are congruent with previous studies demonstrating interoceptive awareness to decline with age (Khalsa et al., 2009). As the core neural substrate of interoceptive processing, the insula also responds to risky decision-making by activating representations of homeostatic states during risk and impacting future decisions (Xue et al., 2010). Specifically, decision making has been viewed as a process of regulating homeostatic states (Paulus, 2011). In addition, previous research has linked heightened interoceptive awareness to panic disorder (Hoffman and Mattis, 2000; Paulus and Stein, 2006). Therefore, exaggerated insular responsivity in adolescents could relate to the decision to engage in risky behaviors and cause adolescents to be more susceptible to mental health issues as well.

Contrary to previous research, however, AD did not exhibit greater activation throughout the ventral striatum than YA and MA. Both AD and YA exhibited greater activation than MA in the right, but not left, ventral striatum. Developmental differences have previously been found in this region (Bjork et al., 2010) as well as in ACC (Eshel et al., 2007). These neural differences are reflective of observed differences in behavior during adolescence compared to adults. Specifically, increased ventral striatum response in adolescents highlights the heightened sensitivity to rewarding properties of stimuli (Chein et al., 2011) while delayed maturation of the ACC correlates with greater risk-taking behaviors during adolescence (Eshel et al., 2007), wherein adolescents do not evaluate potential long-term aversive consequences of rewarding stimuli, thereby failing to inhibit risky behavior (Bjork et al., 2007; Eshel et al., 2007; Doremus-Fitzwater et al., 2010). In addition, AD compared to MA exhibited greater activation in bilateral IFG, a region previously implicated in processing reward and assessing hedonic valence (Craig, 2002; McCabe et al., 2008). This finding, along with greater activation in posterior insula, for AD compared to MA, suggests AD experience the soft touch as more pleasant than MA. Because AD experience the soft touch as more pleasant, as demonstrated by greater activation in the posterior insula, they may need to recruit more IFG resources in order to maintain focus on the task at hand. The type of touch that stimulates both Aβ and CT afferents often occurs during social interactions in our daily life and can provide contextual information about a situation and the emotions and actions of others (Morrison et al., 2011b). Therefore, AD may be more susceptible to social distractions and require more resources from IFG in order to keep focused attention. The lack of a significant difference between groups in the left lentiform nucleus and ACC may be due in part to the smaller sample size of AD. In addition, previous findings were in response to monetary rewards while the task in the present study focused on rewarding touch, which may contribute to neural differences. Coupled together, the differential activation between AD and MA found in the insula during soft touch and ACC during anticipation suggest that adolescents not only experience rewarding stimuli differently but they also engage regulatory structures to a lesser extent than adults, thereby influencing future behaviors differently and resulting in more risk-taking.

There are several limitations to this study. First, the younger adolescents of the sample were restricted to an age range of only 3 years while the adults covered a much larger age range; future studies could expand the age range to cover ages 12–17 for a more comprehensive period of development. Second, data was collected during only 40 repetitions of soft touch total, which may not provide enough power for statistical analyses. Future studies should consider using more repetitions and a longer duration of the soft touch to collect more complete information. Third, groups did not differ in their subjective ratings of the soft touch experience. This could be because VAS ratings were provided after the fMRI session. To differentiate between groups, further investigations might employ a rating scale during the task to more accurately capture subjective ratings in the moment. Fourth, lack of differences in RT across age is another limitation of the study. A more difficult paradigm requiring a higher cognitive load during complex decision-making could be useful in determining whether interoceptive manipulations result in behavioral performance differences between age groups. Lastly, the data presented here is cross-sectional prohibiting conclusions to be drawn about change in neural activation as a function of development. Future studies should employ a longitudinal approach in order to make stronger conclusions about the development of the interoceptive system.

Despite these limitations, these results provide evidence that AD and MA exhibit differential neural responses in brain regions involved in the processing of reward and interoception. The present study is the first to examine these developmental differences in response to positive touch stimulation. Findings provide support for the general hypothesis that the interoceptive system undergoes significant developmental changes. The increased responsivity of the system during adolescents may have profound consequences for a variety of behaviors that emerge during this period. For example, the increased activation in the insular cortex might reflect an enhanced body prediction error signal that results in an increased urge to adjust behavior. This increased urge to act may be adaptive in some circumstances, e.g., to form social relationships, but may also be maladaptive in others, e.g., to impulsively engage in drug use or other risky activities. Future investigations will need to examine whether individual differences during adolescents are able to predict the emergence of different adaptive or maladaptive behaviors. Finally, understanding the contribution interoception and affective touch to the development of various adaptive and maladaptive behaviors may provide information to develop novel interventions based on social touch that are aimed at modulating an individual's responsivity.

### Conflict of interest statement

The authors declare that the research was conducted in the absence of any commercial or financial relationships that could be construed as a potential conflict of interest.
